# Boosting the predictive performance with aqueous solubility dataset curation

**DOI:** 10.1038/s41597-022-01154-3

**Published:** 2022-03-03

**Authors:** Jintao Meng, Peng Chen, Mohamed Wahib, Mingjun Yang, Liangzhen Zheng, Yanjie Wei, Shengzhong Feng, Wei Liu

**Affiliations:** 1 Shenzhen Institutes of Advanced Technology, CAS, Shenzhen, 518000 China; 2 National Institute of Advanced Industrial Science and Technology, Tokyo, Japan; 3 RIKEN Center for Computational Science, Hyogo, Japan; 4 XtalPi. Inc, Shenzhen, 518000 China; 5 National Supercomputer Center in Shenzhen, Shenzhen, 518000 China; 6 Tencent AI Lab, Shenzhen, 518000 China

**Keywords:** Cheminformatics, Scientific data, Computational science

## Abstract

Intrinsic solubility is a critical property in pharmaceutical industry that impacts *in-vivo* bioavailability of small molecule drugs. However, solubility prediction with Artificial Intelligence(AI) are facing insufficient data, poor data quality, and no unified measurements for AI and physics-based approaches. We collect 7 aqueous solubility datasets, and present a dataset curation workflow. Evaluating the curated data with two expanded deep learning methods, improved RMSE scores on all curated thermodynamic datasets are observed. We also compare expanded *Chemprop* enhanced with curated data and state-of-art physics-based approach using pearson and spearman correlation coefficients. A similar performance on pearson with 0.930 and spearman with 0.947 from expanded *Chemprop* is achieved. A steadily improved pearson and spearman values with increasing data points are also illustrated. Besides that, the computation advantage of AI models enables quick evaluation of a large set of molecules during the hit identification or lead optimization stages, which helps further decision making within the time cycle at drug discovery stage.

## Introduction

Aqueous solubility is one of the critical factors defining the bio-availability of orally administrated drugs. Reportedly, over 75% of oral drug development candidates have a low solubility based on the Bio-pharmaceutics Classification System (BCS)0^1,2^. To tackle this challenge, researchers are focusing on drug solubility improvements with both physics-based Quantum Mechanics-Quantitative Structure Property Relationships (QM-QSPR) approaches^3–6^ and data-driven artificial intelligence (AI) methods^7–11^.

The development of **QM-QSPR approaches** provides a large number of computational methods for aqueous solubility prediction starting from a molecular structure^3–6^. The majority of these methods try to explore fundamental physics-based rules with a sublimation thermodynamic cycle solubility approach^2,12^ on crystalline drug-like molecules. This approach is an interplay between crystal packing and molecular hydration free energy contributions^12–15^. With this approach, a crystal packing contribution to the drug solubility typically requires a sublimation energy estimation from crystal lattice calculations^12–14^, molecular dynamics simulations^16^, or QSPR statistical models^15,17^. The free energy of solvation may be estimated by a variety of approaches, including QSPR models, monte carlo simulations, and QM-based methods^18^. Recently, a study of guiding lead optimization^2^ was proposed. It explicitly describes the solid-state contribution, and the superior performance of the QM-based thermodynamic cycle approach is demonstrated in the optimization of two pharmaceutical series. The main limitations of the physics-based QM-QSPR approaches are the large compute requirements and long run time. For example, guiding lead optimization^2^ relies on crystal structure prediction calculations^19^, which may require several days on a powerful cloud infrastructure consisting of millions of CPU cores.

Early AI-based approaches for solubility prediction involve the application of logistic regression^7^, random forests^8^ and convolutional neural networks^9^ to expert-engineered descriptors^10,11^ or molecular fingerprints such as the Dragon descriptors or Morgan (ECFP) fingerprints^20–22^. Their predictive accuracy or equivalent root mean square errors (RMSE) is limited to 0.7–1.0 log. Recent research efforts are focused on graph learning^23–26^ of the underlying topology of molecule structure using SMILES strings^27^. Such models extract their own expert features directly from atoms and edges, and embed them with graph convolutional networks. An experiment in MoleculeNet^23^ on solubility prediction of ESOL dataset ranks Message Passing Neural Network (MPNN) as the best graph learning model with a predictive accuracy of 0.58 among other graph models, such as WEAVE^28^ and GraphConv^26^. Chemprop^24^ which embeds molecule-level features and extends the Message Passing Neural Network (MPNN) with Directed MPNN, further improves the predictive accuracy on ESOL to 0.56. *AttentiveFP*^29^ is the first work applying attention mechanism with a graph neural network and reports the lowest accuracy of 0.503 on ESOL dataset. These deep learning based approaches are trying to model complex physicochemical properties with a QSPR statistical approach, however their flexibility and capacity of capturing those complex relationships are still bounded by the availability of high quality data^30–32^.

The measurement and dataset diversity gap between the AI-based and QM-QSPR approaches are two critical issues hindering the research on combining these two approaches. For AI-based approaches in particular, different papers evaluate their work on different datasets, using different workflows, or even with different measurements. In most cases, this becomes the first obstacle preventing readers from objectively distinguishing the viability of the proposed AI-approaches. More importantly, to the authors knowledge, no previous work conducted any comparison to evaluate both AI-based and QM-QSPR approaches under the some measurements with an open available dataset. This situation also inhibits any quantitative analysis from exploring the advantages and disadvantages of these two approaches, and the possibilities of combining them to achieve additional progress.

In term of data curation methodologies, previously Eriksson’s work published in 2003^33^, takes prepossessing techniques (scaling and centering), data correction, and transformations to improve the regression model’s performance on Quantitative Structure-Activity Relationship (QSAR). There are three different points between our work and Eriksson’s work. Firstly, Our work is focused specially on solubility instead of QSAR. The data correction using signal correction actually cannot work on our dataset, as there are no relationship between the solubility value and undesired variation arising from light-scattering effects, baseline drift, nonlinearities, and so forth. Secondly, our work is exploring data curation methodology for nonlinear deep learning model using graph neural networks, whereas Eriksson’s work^33^ is targeting on linear regression model. last, our work on is focused on data curation methodology itself. Eriksson’s work needs the prepossessing techniques (scaling and centering) and transformations steps to avoid large influence on the model and dominating over the other measurements from unbalanced data composition. However, these problems has been resolved in our work by using scaffold data partition. Our work is the only work focused on inter-dataset redundancy and intra-dataset redundancy, it is a novel technique not yet presented by any previous work.

To conclude the above discussion, solubility prediction with AI-based methods still face the following three challenges:

1. The volume of training data in previous works, such as the ESOL dataset, is limited. Training and evaluation on these small datasets do not necessarily offer good performance for our problems. These datasets are also insufficient for sophisticated models attempting to learn massive physical-chemical rules and converge to a stable state.
2. Data curation methods or tools for low-quality aqueous solubility data are still lacking. Directly training on data with poor quality may affect the predictive accuracy.
3. None of the previous studies pose a comparison of the predictive accuracy between leading deep learning and state-of-the-art QM-QSPR approaches. Analyzing and determining the advantages and disadvantages of deep learning methods in comparison with the QM-QSPR approaches is also critical but difficult to achieve.

To resolve the above issues and refine the research problem of solubility prediction for AI, our contributions are threefold:

1. The first large-scale dataset for AI research on aqueous solubility is collected. This dataset contains seven aqueous solubility datasets including both thermodynamic and kinetic data. The number of records in these datasets ranges between a few thousand to several hundreds of thousands.
2. This work is the first to improve the aqueous solubility predictive accuracy with a data curation method. We present a data curation workflow of filtering, evaluating and clustering. This workflow adds solubility quality to each record and curates records sharing similar solubility among different datasets. We also expand two leading deep learning methods, i.e., *Chemprop*^24^ and *AttentiveFP*^29^, to support data quality during the training and evaluation process. Using these expansions of the *Chemprop* and *AttentiveFP* deep learning methods, improved predictive accuracy is observed on all thermodynamic datasets.
3. This work is also the first to compare deep learning and QM-QSPR approaches using the pearson and spearman’s rank-order correlation coefficients by predicting four pharmaceutical series of 48 molecules. Abramov’s guiding lead optimization and weighted *Chemprop* are selected as the representatives for both. By predicting the first two pharmaceutical series of 31 molecules, Abramov’s approach demonstrates a pearson correlation coefficient *r*^2^ of 0.905 and spearman’s rank-order correlation coefficient *R*_*s*_ of 0.967. Weighted *Chemprop* (expanded to support the high data quality) is trained on the curated dataset yielding improvement in its *r*^2^ and *R*_*s*_ values. It increases steadily with the increase in training data volume and further achieves comparable performance on *r*^2^ with 0.930 and *R*_*s*_ with 0.947. In comparison with Abramov’s approach, which requires a large compute resources, predicting the thousands of target compounds with deep learning approach takes only seconds on a common desktop computer.

The rest of this paper is organized as follows. The collection and description of seven datasets, together with our data curation workflow, are illustrated in Section methods. Section results compares the deep learning and QM-QSPR approaches and then discusses the benefits of data curation. Section discussion explains the innovations and contributions this work make towards molecule property prediction.

## Methods

### Datasets

We collected molecules labeled with aqueous solubility from publicly available databases or datasets provided by previous papers, resulting in the 7 datasets shown in Table 1. Among them, the first three datasets were evaluated by previous papers^11,23,24,34,35^ but are limited in number of samples or records, while the last four datasets have larger number of samples with poor data quality. We also include both thermodynamic and kinetic datasets; the first six are the thermodynamic datasets, while the last is the kinetic set^36^.

**Table 1:** Statistical information of the number of records in the 7 collected datasets.

Dataset	No. of Records in			Weights	Additional Columns of Org Dataset
	Org	Cln	Cure		
AQUA	1311	1311	1354	1.0	
PHYS	2010	2001	2001	1.0	star_flag
ESOL	1128	1116	1157	1.0	
OCHEM	6525	4218	3766	0.85	
AQSOL	9982	8701	9061	0.4	group
CHEMBL	30099	30099	28675	0.8	comment
KINECT	164273	82057	81935	—	temperature, pH value

“Org” is the original dataset, “Cln” denotes the dataset after Data Filtering, “Cure” is the dataset after Data Curation using the clustering algorithm across multiple datasets, “Weights” denotes the assigned weights for each dataset to identify the dataset quality, and “Additional Columns of Org Dataset” includes special properties reserved by some of the datasets.

Table 1 demonstrates the statistical information of each dataset. Every dataset is processed separately to have the same standardized form. The data extraction process and standardization methods applied for each dataset are described below.

- AQUA. This dataset was taken from the work of Huuskonen^34^ and Tetko^11^, with 1311 records on 1307 molecules downloaded from the ALOGPS homepage at http://146.107.217.178/lab/alogps/logs.txt. The experimental aqueous solubility value is measured between 20–25 °C and obtained partly from the AQUASOL database of the University of Arizona and SCR’s PHYSPROP database.
- PHYS. This dataset is a curated PHYSPROP database consisting of a collection of datasets in SDF format. An automated KNIME workflow^37^ is used to curate and correct errors in the structure and identity of chemicals using the publicly available PHYSPROP datasets. Here, we extract 2024 molecules with a water solubility (WS) endpoint. The quality of each record is measured with stars from 1 to 5; thus, the data quality property of “STAR_FLAG” is reserved, and finally 2010 records is reserved.
- ESOL. The original ESOL dataset, containing 1144 records, was first used by^35^, and then its verified version was evaluated in^23,24^. We downloaded its verified version with 1128 records from Chemprop’s repository https://github.com/Chemprop/Chemprop as our ESOL dataset to keep it consistent with previous works^23,24^.
- OCHEM. This dataset is taken from the OCHEM database of WS at https://ochem.eu/. We reserve 6525 rows from 36,450 records by selecting molecules with the dataset type “Training” to reserve molecules with experimental solubility values.
- AQSOL. This dataset^38^ combines 9 datasets, including the AQUA and ESOL datasets. A preprocessing step is used to filter this dataset by merging repetitive molecules, with 9982 records remaining. According to the number of occurrences in the 9 original datasets, a new property called “group” is added to this dataset by using a classification strategy that can group this dataset into 5 groups. We keep “group” in this dataset for further assignment of the weights to identify the data quality of each record.
- CHEMBL. This dataset is extracted from CHEMBL’s activity database, which includes 15,996,368 records at https://www.ebi.ac.uk/chembl. We filter this dataset with the assay type “physicochemical” and then select 40,520 records with the standard type “Solubility” or “solubility” as our dataset. Several different units are used for the aqueous solubility measurement, such as nM, ug/mL, and ug.mL-1. All the units are converted to standard “LogS” units. We find 4,543 records are kinetic solubility data and 17 records are using oil as the solvent. Thus we further removed all these records to clean our CHEMBL dataset from kinetic solubility data. Finally 30,099 valid records are reserved. In addition, the column “Comment” describing the temperature and pH of the experiment is kept for later weight assignment on the data quality of each record.
- KINECT. This dataset is taken from the OCHEM database of WS based on the Kinect technology at https://ochem.eu/. 164,273 records described in SDF format are extracted and collected into this dataset. In addition, the columns of properties “SMILES”, “LogS value”, “pH value” and “Temperature” are also extracted and reserved for quality weight assignment.

### Data curation

Due to the various experiment environments, workflows and non-unique identifications, the records in the aqueous solubility datasets are repetitive, erroneous or even contradictory to each other^37,38^. Note that molecules with the same SMILES may be different tautomers^39^, and thus have different solubility value. As SMILES can not distinguish tautomers thus we just keep them into different records but with different solubility value. We merge the two records only when the difference of these two value is less than 0.5.

The development of reliable data-driven deep learning models, however, may be hindered by uncertainties and disagreements in these repetitive records, which are obtained from many disparate data sources. Training data with systematic errors from different experimental methodologies potentially limit the predictive accuracy of deep learning models. To improve the predictive accuracy of deep learning methods and achieve a better generalization ability from low-quality and confusing data, a curation method delivering high-quality data, balanced on substructure classes and sufficient in terms of the data volume, is vitally important.

**Fig. 1:**
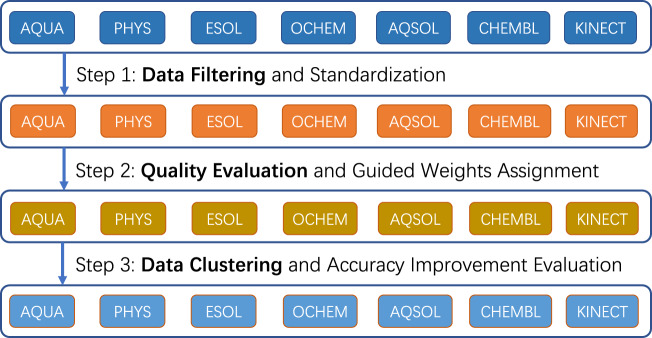
The data curation workflow of filtering, evaluating, and clustering on the 7 collected datasets.

We present a data curation workflow of filtering, evaluating and clustering for the above 7 datasets as illustrated in Fig. 1. The workflow tries to improve the dataset quality by data filtering, a quality evaluation and then cross-dataset correction among different datasets with a clustering algorithm. Finally, an evaluation with two leading deep learning methods, i.e., *Chemprop* and *AttentiveFP*, demonstrates the benefits of this workflow in predictive accuracy improvement based on the RMSE over all thermodynamic datasets.

#### Data filtering

To resolve the standardization of the molecule expressions, uncertainties from various experiment environments, and weight bias from repetitive data, the data filtering strategy is proposed with the following three steps: SMILES standardization, experiment environmental control, and repetitive record normalization.

- **SMILES standardization** First, each molecule has only one unique SMILES expression in different databases. MolVS (described at https://molvs.readthedocs.io/en/latest/) is used to standardize all chemical structures and maintain one unique standard SMILES for each molecule. Any molecule that fails to pass our standardization procedure is removed from the dataset.
- **Experiment environmental control** Second, we target the aqueous solubility prediction of small molecules in drug design. Thus, the experiment environment of molecules with temperatures of 25 ± 5 °C and pH values of 7 ± 1 are highly valued; any records beyond our scope are ranked low or even removed. Any molecule used for drug design should be poison-free. For this reason, molecules with heavy metals such as “U, Ge, Pr, La, Dy, Ti, Zr, Rh, Lu, Mo, Sm, Sb, Nd, Gd, Cd, Ce, In, Pt, Sb, As, Ir, Ba, B, Hg, Se, Sn, Ti, Fe, Si, Al, Bi, Pb, Pd, Ag, Au, Cu, Pt, Co, Ni, Ru, Mg, Zn, Mn, Cr, Ca, K, Li” are filtered from all datasets. “SF5,SF6” are also cleaned, as they are rarely used in drug design.
- **Repetitive record normalization** Third, some datasets contain repetitive molecules with equal or different solubility values. According to the frequency of occurrence, repetitive record normalization is carried out to assign weights to each molecule, with a total weighted value of 1.0, to prevent those molecules with repetitive values from gaining larger parameter update weights during the model training process.

The number of data records before and after our data filtering is presented in Table 1. For each cleaned dataset, the available information in terms of the name, description, and column type are presented in Table 2. In the end, 1311, 2001, 1116, 4218, 8701, 30,099, and 82,057 records are in cleaned AQUA, PHYS, ESOL, OCHEM, AQSOL, CHEMBL, and KINECT datasets, respectively.

**Table 2:** List of information for all cleaned and curated datasets in terms of the name, description, and type of each column.

Column Name	Description	Type
Smiles	SMILES representation of compound	String
LogS	Experimental aqueous solubility value (LogS)	String
Weight	weighted quality score in [0, 1]	Float

#### Quality evaluation

Quality evaluation is performed to analyze, evaluate and assign each dataset with an appropriate weight to identify its quality. We first analyze the molecule redundancy among different datasets with identical or different solubility values. Then, we expand *Chemprop* and *AttentiveFP* to support the data quality weights and refer to them as weighted *Chemprop* and weighted *AttentiveFP*. weighted *Chemprop* is used to evaluate each dataset’s predictive accuracy (measured in RMSE) to identify the dataset quality. Finally, each dataset is assigned a weight indicating its data quality.

The existence of data redundancy in repetitive records generate bias in the model training process and evaluation metric. Several data redundancies can be found both within and among the datasets. These data redundancies can be classified into two classes: those in which a given molecule was found in two records with identical solubility values and those in which a given molecule was found in two records with different solubility values. Here, we define solubility values with a 0.01 LogS unit difference between two records as identical. Notably, these redundancies can be found in two records from a single dataset or from two different datasets. The former case is normalized first by repetitive record normalization, as discussed in the previous subsection; thus, there is no molecules sharing the same value occur twice in a single dataset.

**Fig. 2:**
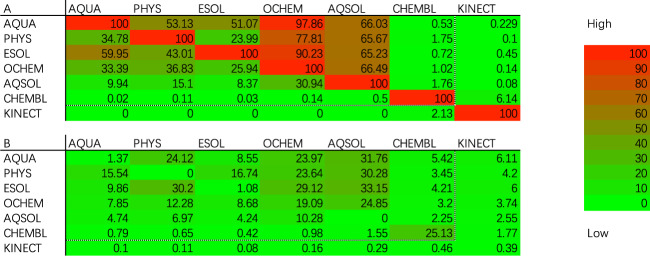
Redundancy matrices showing the percentage of repetitive molecules between two datasets. The upper table *A*_*ij*_ summarizes the percentages of molecules with the same solubility values, and the lower table *B*_*ij*_ describes the percentages of molecules with different solubility values.

With the above definitions, two redundancy matrices are collected, as presented in Fig. 2, where the percentages of repetitive molecules with the same and different solubility values are presented in the upper and lower tables, respectively. The rows or columns of these two tables represent the corresponding datasets. The percentage of repetitive molecules with the same solubility value between two datasets *i* and *j* is represented as *A*_*ij*_, and that with different solubility values is represented as *B*_*ij*_. For example, *A*_*ESOL,PHYS*_ = 43.01 indicates that 43.01% of the records (one molecule can have multiple records) in the ESOL dataset can be found in the PHYS dataset with the same solubility value. As another example, *B*_*CEHMBL,CHEMBL*_ = 25.13 reveals that 25.13% of the records in the CHEMBL dataset can be found sharing the same molecule but with different solubility values in the same dataset. Note that the two redundancy matrices in Fig. 2 are not symmetric for different dataset sizes. The sum of *A*_*ij*_ and *B*_*ij*_ for corresponding datasets *i* and *j* can be beyond 100%, as given a record from dataset *i*, a molecule in dataset *j* can have multiple records and thus can share both the same and different solubility values with the same molecule in other datasets.

A preliminary analysis of the records in and between datasets reveals the potential value of data curation. In the upper table of Fig. 2, approximately half of the records in AQUA, PHYS, and ESOL share the same solubility values. Approximately 77–98% and 66% of the records in AQUA, PHYS and ESOL are contained in OCHEM and AQSOL, respectively. In the lower table, 9–30% of the records share different solubility values among AQUA, PHYS and ESOL. More than 24% and 30% of the records in these three datasets share different solubility values. CHEMBL has its own speciality. Both tables confirm that CHEMBL contains few records from other datasets, and the lower table confirms that one-quarter of the records in CHEMBL have diverse solubility values. Our intuition on data curation is to make use of the above record redundancies. In practice, a record for a given molecule with the same solubility value in more than one dataset can help us to improve the confidence regarding its data quality. Likewise, a record with different values among datasets can decrease the confidence with regard to its data quality. This is the fundamental difference between our work and a previous work^38^ as a result of selecting those records with multiple occurrences. Thus, the percentages of both inter-dataset and intra-dataset record redundancies will determine the effectiveness of our data curation method.

To analyze the quality of each dataset, one of the leading graph learning methods named as *Chemprop* is selected to evaluate all 7 datasets, with the predictive accuracy used as a reference. Both random and scaffold splitting are used in this evaluation. Here random splitting randomly splits samples into training, validation, and test three subsets. Scaffold^40^ splitting splits the samples based on their two-dimensional structural frameworks as implemented in RDKit. Scaffold splitting is a useful way of organizing the structural data to group the atoms of each drug molecule into ring, linker, framework, and side chain atoms. Considering that random splitting of molecular data isn’t always best for evaluating machine learning methods. Scaffold splitting is also applied in our evaluation. For the original datasets, we train each dataset with *Chemprop* using both random and scaffold data partition ratios of [0.8, 0.1, 0.1] for training, testing, and evaluation. Moreover, we ensemble 5 models to improve the model accuracy and record the average RMSE value and its confidence intervals by running each ensembled model 8 times. The RMSE value of the original dataset is recorded and collected in the third column of Table 3. Multiple different solubility values for a given molecule among the datasets are normalized on weights according to the statistical distribution of the molecule determined by the previously discussed data filtering process. However, *Chemprop* does not support weighted quality scores for records in a cleaned dataset. Thus, we expand the training and evaluation codes of *Chemprop* to support training over weighted records and rename it weighted *Chemprop*. As a result, a record with higher weighted quality has a contributes to a larger extent in the parameter update, whereas records with lower weights have a smaller effect. Note that when dataset contains no weights, weighted *Chemprop* treats each record equally and acts the same as *Chemprop*. Trained on these 7 cleaned datasets, the corresponding prediction accuracy measured with the RMSE is collected in the fourth column of Table 3.

**Table 3:** The collected RMSE and confidence intervals of *Chemprop* or weighted *Chemprop* trained on the 7 datasets.

Split Type	Dataset	RMSE & Confidence Intervals		
		Org	Cln	Cure
Random	AQUA	0.573 ± 0.037	0.583 ± 0.057	0.536 ± 0.042
	PHYSP	0.550 ± 0.026	0.600 ± 0.032	0.515 ± 0.018
	ESOL	0.596 ± 0.075	0.619 ± 0.044	0.512 ± 0.047
	OCHEM	0.548 ± 0.024	0.639 ± 0.044	0.522 ± 0.017
	AQSOL	1.023 ± 0.035	0.820 ± 0.036	0.518 ± 0.022
	CHEMBL	0.917 ± 0.017	0.811 ± 0.016	0.499 ± 0.011
	KINECT	0.401 ± 0.003	0.431 ± 0.003	0.432 ± 0.003
Scaffold	AQUA	0.850 ± 0.086	0.849 ± 0.075	0.697 ± 0.043
	PHYS	0.833 ± 0.058	0.813 ± 0.115	0.691 ± 0.092
	ESOL	0.854 ± 0.097	0.808 ± 0.090	0.711 ± 0.073
	OCHEM	0.847 ± 0.067	0.808 ± 0.075	0.695 ± 0.061
	AQSOL	1.073 ± 0.062	0.968 ± 0.045	0.596 ± 0.033
	CHEMBL	1.040 ± 0.038	0.900 ± 0.049	0.555 ± 0.031
	KINECT	0.433 ± 0.015	0.461 ± 0.008	0.460 ± 0.008

The data partition strategies include both random and scaffold strategies. Five models are ensembled to improve the model accuracy. We average the RMSE by running each model 8 times and then calculate the corresponding confidence interval. The original *Chemprop* is used on “Org” dataset, and the weighted *Chemprop* is applied on both “Cln” and “Cure” datasets.

Root Mean Square Error (RMSE) is a standard way to measure the error of a model in predicting quantitative data, detailed definition of RMSE is presented at https://en.wikipedia.org/wiki/Root-mean-square_deviation. Assume that there are *n* records in the test subsets, formally the RMSE of this test subsets is defined as follows:

$$RMSE=\sqrt{\mathop{\sum }\limits_{i=0}^{n-1}\frac{{({\bar{y}}_{i}-{y}_{i})}^{2}}{n}}$$

Here, ${\bar{y}}_{0},{\bar{y}}_{1},\ldots ,{\bar{y}}_{n-1}$ are predicted values. *y*_0_, *y*_1_, …, *y*_*n*−1_ are observed values. *n* is the number of records in the test subsets. As all the clean and cure dataset contains quality weights, we must update RMSE for both evaluation and test subset to use the weighted records during our training process. Thus we updated the evaluation metric with weighted records, the definition of our weighted RMSE is described as below. Assume that there are *n* records in the test subsets, formally the weighted RMSE of this test subsets is defined as follows:

$$RMSE=\sqrt{\mathop{\sum }\limits_{i=0}^{n-1}\frac{{w}_{i}\ast {({\bar{y}}_{i}-{y}_{i})}^{2}}{n}}$$

Here, ${\bar{y}}_{0},{\bar{y}}_{1},\ldots ,{\bar{y}}_{n-1}$ are predicted values. *y*_0_, *y*_1_, …, *y*_*n*−1_ are observed values. *w*_0_, *w*_1_, …, *w*_*n*−1_ are quality weights of each records. *n* is the number of records in the test subsets. In the original dataset, there are no quality weights, we treat each record with unit weights by default to calculate its weighted RMSE. As you can see, the weighted RMSE will be same as RMSE when using unit weights for original dataset. Thus the weighted RMSE is a comparable metric across the original, clean and cure datasets, and in this paper we use RMSE for simple to denote “weighted RMSE” for curated datasets. The original *Chemprop* is used on “Org” dataset, and the weighted *Chemprop* is applied on both “Cln” and “Cure” datasets.

According to Table 3, the six thermodynamic datasets can be split into two groups. The first group includes AQUA, PHYS, ESOL and OCHEM, and the second group includes AQSOL and CHEMBL. The datasets in the first group have smaller populations and relatively lower RMSE values; we denote the datasets in this group as high-quality datasets. The second group has massive records and higher RMSE values in both the original and cleaned datasets; thus, the two datasets are regarded as low-quality datasets. Due to the change in the evaluation metric with the weighted records in the high-quality datasets and KINECT dataset, the predictive accuracy of each clean dataset has a 10% increase in the RMSE using a random partition compared with the original dataset. At the same time, as we take “group” and “comment” as references to carry out weight assignment for each record in the low-quality datasets, weighted *Chemprop* learns over the quality weights after repetitive record normalization and then benefits from a slightly decrease in predictive accuracy (lower is better).

With the above analysis, we can initialize and assign a quality weight for each dataset. The assigned quality weight is used for data curation in the following section. The assigned weights are distributed in [0, 1], with a value close to 1 indicating high data quality. The assigned weights for these six thermodynamic datasets are listed in the fifth column of Table 1. The KINECT dataset is the only kinetic-based dataset; thus, no weighted quality is set. The weights in Table 1 are presented as an example to show a relative ranking in terms of the data quality among the different datasets, and the specific weight for each dataset can still be adjusted. Searching for and evaluating a better weight assignment require extremely large compute power, e.g., one round of evaluation generating all the data in Table 3 costs approximately two weeks using 1200 compute nodes (38,200 cores and 4800 GPU accelerators) in the National Supercomputer Center in Shenzhen. Therefore, we estimate the weights in Table 1 from our first intuition and then calculate the corresponding predictive accuracy results in Table 3.

#### Data clustering

This work is the first to curate data using inter-dataset redundancy and intra-dataset redundancy. Three curation guidelines are followed to take advantage of these datasets with potential redundancy: **❶** A dataset with a higher quality weight can be used to curate a dataset with a lower weight. **❷** The final quality weight of a record from a dataset can be calculated by multiplying the weight of the record itself by the assigned weight of the dataset. **❸** Records with similar solubility values for a given molecule can be merged by averaging their solubility values over their weights.

**Fig. 3:**
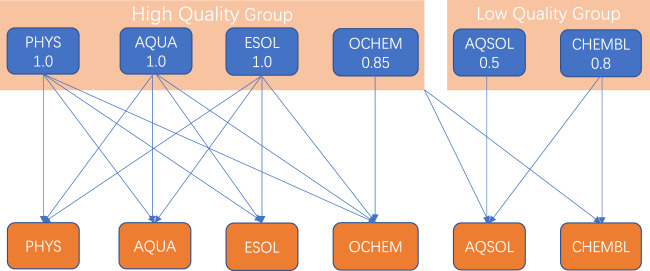
Data curation schedule for the 6 thermodynamic datasets. The datasets are divided into 2 groups: high quality and low quality groups. Two curation operations, i.e., inter-group curation and intra-group curation, are illustrated. The feasible curation operations for each dataset are denoted by the lines. For example, AQUA can be curated with the AQUA, PHYS, and ESOL datasets, and AQSOL can be curated with all dataset in high quality group, and CHEMBL.

First, a curation schedule following guideline **❶** is designed, as demonstrated in Fig. 3. Previously, we divided the six thermodynamic datasets into two groups: a high- and low-quality group. As illustrated in Fig. 3, one can curate a dataset with other datasets in the same group with higher or equal weights, which is denoted as inter-group curation. A dataset in the high-quality group can be used to curate a dataset in the low-quality group, which we refer to as intra-group curation. No other operations are allowed.

Second, a record clustering and curation workflow is adapted to implement guideline **❷**. Given a set of *n* cleaned datasets *D*[*i*], each records is initialized with our workflow aims to curate *D*[*n* − 1] with datasets *D*[0], …, *D*[*n* − 2]. Our curation workflow contains three steps: (1) We merge all input datasets *D*[*i*] and reserve all the records with the same compound contained by dataset *D*[*n* − 1] as a new dataset *T*. (2) For each molecule with multiple solubility values, a partial clustering algorithm, illustrated in Algorithm 1, is adopted to merge these records. Then, we update the solubility values and weights with the equation listed by line 5 and line 6 in algorithm 1 for each molecule in *T*. (3) We accumulate the total weights for each molecule and truncate the maximum total weights with a given *threshold*. Then, the weights for each record are normalized in *T*. By adjusting *threshold*, those molecules occurring in multiple datasets and thus accumulating high total weights larger than *threshold* become highly valued, and those molecules with total weights less than *threshold* become devalued.

Third, the partial clustering algorithm mentioned above is designed, as presented in Algorithm 1, to cure the records following guideline **❸**. In each while loop, the two closest solubility values for a given molecule are selected and merged if their difference is less than a given parameter *d*. The two records are merged by averaging their solubility values over their weights, and their two weights are summed as the new quality weight. If the difference between the two closest values is larger than *d*, the while loop ends and the merged records are updated as the new record. For the parameter *d*, we recommend using 0.5 as suggested in^11^.

**Algorithm 1** Partial Clustering Algorithm.

The above workflow is developed and open-sourced in our repository

https://github.com/Mengjintao/Chemprop. The seven curated datasets are collected by applying this workflow, and then weighted *Chemprop* is trained on these datasets. For the best ensembled models, the solubility prediction accuracy values measured in terms of the RMSE are summarized in Table 3. Here the lowest RMSE value is recorded for KINECT, being as low as 0.432 (with a confidence interval of 0.003). ESOL is a widely used benchmark in previous research, and its RMSE score decreases from 0.596 (0.56 reported by *Chemprop* with Bayesian optimization^24^) to 0.512, i.e., a 0.084 LogS unit decline after data curation. On other datasets with a random data partition, the RMSE values of weighted *Chemprop* benefit from a dramatic decline of 0.037, 0.035, 0.026, 0.505, and 0.418, respectively, on the curated AQUA, PHYS, OCHEM, AQSOL, and CHEMBL datasets. With scaffold data partition, the RMSE values decreasing by 0.153, 0.142, 0.152, 0.477, and 0.485, respectively. The model trained on the curated KINECT dataset, however, records an increase in the RMSE value under both random and scaffold data partition, as the KINECT dataset is the only set of Kinect solubility data; hence, no other dataset can be used to curate this dataset. Moreover, the limited inter-dataset redundancy demonstrated in Fig. 2 on the KINECT dataset also restricts our curation benefits. Even with the above limitations, KINECT dataset still contributes the lowest RMSE score among all datasets with both Random and scaffold data partition.

In addition to *Chemprop*, we include another recently developed deep learning method named *AttentiveFP* in our evaluation. *AttentiveFP* follows a traditional graph learning mechanism and allows non-local effects at the intra-molecular level by applying a graph attention mechanism with multiple GRU layers. We also expand the code of *AttentiveFP* to support data quality weights during training and evaluation. The GitHub repository of weighted AttentiveFP is https://github.com/Mengjintao/AttentiveFP. An evaluation workflow similar to that of *Chemprop* is used, ensembling multiple *AttentiveFP* models in several folds. The RMSE values and confidence intervals of *AttentiveFP* on all 7 datasets are collected in Table 4. A similar trend with a decreasing RMSE value is illustrated in Table 4. For example, *AttentiveFP* trained on the curated AQUA, PHYS, ESOL, OCHEM, and AQSOL datasets achieves 0.067, 0.095, 0.03, 0.043, and 0.242 unit log decreases in the RMSE compared with the original dataset using a scaffold data partition.

**Table 4:** The collected RMSE and confidence intervals of *AttentiveFP*when trained on the 7 datasets^29^.

Split Type	Dataset	RMSE & Confidence Intervals		
		Org	Cln	Cure
Random	AQUA	0.616 ± 0.027	0.639 ± 0.014	0.579 ± 0.020
	PHYS	0.649 ± 0.019	0.643 ± 0.013	0.551 ± 0.024
	ESOL	0.642 ± 0.017	0.641 ± 0.025	0.594 ± 0.022
	OCHEM	0.6018 ± 0.012	0.651 ± 0.020	0.6016 ± 0.010
	AQSOL	0.826 ± 0.027	0.760 ± 0.012	0.593 ± 0.004
Scaffold	AQUA	0.743 ± 0.038	0.747 ± 0.031	0.676 ± 0.038
	PHYS	0.782 ± 0.037	0.789 ± 0.037	0.687 ± 0.038
	ESOL	0.761 ± 0.048	0.801 ± 0.043	0.731 ± 0.073
	OCHEM	0.746 ± 0.011	0.779 ± 0.019	0.703 ± 0.016
	AQSOL	0.872 ± 0.017	0.842 ± 0.019	0.630 ± 0.008

The data partition strategies include both random and scaffold partitioning, and the partition ratio is [0.8, 0.1, 0.1] for training, testing, and evaluation. In this experiment, 5 models are ensembled 8 times to average the RMSE values and calculate the corresponding confidence interval. Because *AttentiveFP*is time consuming on a very large dataset, the CHEMBL and KINECT datasets are not recorded, as their training times are longer than 150 hours. The original AttentiveFP is used on “Org” dataset, and The weighted AttentiveFP is applied on both “Cln” and “Cure” datasets.

All the evaluations demonstrated above in Tables 3, 4 employ hyperparameter optimization with a grid search approach. The grid search approach randomly selects 108 parameter combinations on five key parameters, and the lowest RMSE value is recorded. A larger search space may decrease the RMSE value further but will not change the trend demonstrated in Tables 3, 4; thus, we keep the same number of parameter combinations in our search space during the entire work and do not enlarge the search space to reduce the training time and computing resources.

## Results

The disparate statistical measurement and high quality datasets are the main obstacles to making an objective comparison between deep learning and QM-QSPR approaches, in terms of solubility prediction. To conduct a comparison, a dataset of 48 molecules is gathered from several previous works^2,41,42^. This dataset includes four pharmaceutical series of 48 molecules, and none are contained in the 7 collected datasets. pearson and spearman’s rank-order correlation coefficients are used to evaluate the performances of the deep learning and QM-QSPR approaches.

The correlation coefficients of the predicted and observed values are the main concern for lead optimization in compound design. The thermodynamic cycle solubility approach is a fundamental theory used in the QM-QSPR approaches. In this approach, the log scale of the aqueous solubility value is linearly related to the sublimation and hydration free energies. QM-QSPR approaches mainly focus on searching for extremely accurate methods to calculate the sublimation and hydration free energies using a physics-based simulation at the cost of enormous supercomputing power or quantum computation. Thus, instead of predicting the absolute solubility values, the main goal of the QM-QSPR approaches is to evaluate the correlation coefficient of the solubility value with its two energy factors and then apply it in lead optimization. Two measurements are recommended by one state-of-the-art work^2^ to evaluate the correlation coefficient: the square of the pearson correlation coefficient *r*^2^ and spearman’s rank-order correlation coefficient *R*_*S*_. The equation for the pearson correlation coefficient *r* is

1 $$r=\frac{{\sum }_{i=0}^{n-1}({x}_{i}-\bar{x})({y}_{i}-\bar{y})}{\sqrt{{\sum }_{i=0}^{n-1}{({x}_{i}-\bar{x})}^{2}}\sqrt{{\sum }_{i=0}^{n-1}{({y}_{i}-\bar{y})}^{2}}}.$$

Here, *x* is the vector of the predicted value, *y* is the vector of the true value, and $\bar{x}$ and $\bar{y}$ are the average values of *x* and *y*, respectively. When *r*^2^ equal to 1, this indicates a perfect linear correlation between the observed and predicted solubility values. spearman’s rank-order correlation coefficient *R*_*S*_ can be calculated as

2 $${R}_{S}=1-6\mathop{\sum }\limits_{i=0}^{n-1}({d}_{i}^{2})/n({n}^{2}-1),$$

Where *d*_*i*_ is the difference between the ranks of the measured and predicted solubilities of molecule *i*. Here, *R*_*S*_ equal to 1 indicates a perfect ranking of the predicted solubility values.

**Fig. 4:**
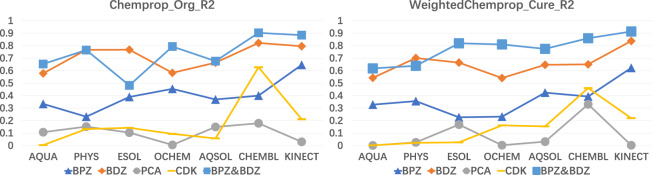
Comparison of *r*^2^ values for ensembled models with the best RMSE scores in Table 3 for *Chemprop* (left figure) or weighted *Chemprop* (right figure) when predicting 48 molecules.

**Fig. 5:**
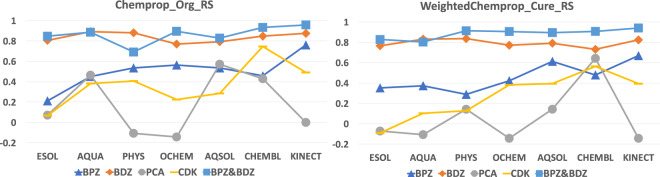
Comparison of *R*_*s*_ values on ensembled models with the best RMSE scores in Table 3 for *Chemprop* (left figure) or weighted *Chemprop* (right figure) when predicting 48 molecules.

We compare the deep learning and QM-QSPR approaches on *r*^2^ and *R*_*S*_ with the evaluation dataset of 48 molecules. The ensembled models resulting in the best RMSE value in Tables 3, 4 are used to predict the evaluation dataset with weighted *Chemprop* (Chemprop expanded to support data quality). In this evaluation dataset, 12 molecules of Benzoylphenylurea (BPU) derivatives and 19 molecules of Benzodiazepin (BDZ) derivatives comprise the first 31 molecules^2^. Seven molecules with selective Cyclin-Dependent Kinase 12 (CDK) inhibitors^42^ and 10 molecules of Pyrazole and Cyanopyrrole Analogs (PCAs) comprise the last 17 molecules^41^. We collect the statistical results of *r*^2^ and *R*_*S*_ on these 48 molecules for weighted *Chemprop* and plot them in Figs. 4, 5, respectively. Note that the *r*^2^ values of the QM-QSPR approach proposed by^2^ are 0.79, 0.83, and 0.905 on the BPU, BDZ, and BPU&BDZ datasets, respectively. They also report the *R*_*s*_ score on the BPU, BDZ, and BPU&BDZ datasets to be 0.87, 0.90 and 0.967 respectively. Currently, no statistical results have been given on PCAs and CDK inhibitors by any of the QM-QSPR approaches.

In Fig. 4, the *r*^2^ curves of weighted *Chemprop* on BPU, BDZ, and BPU&BDZ increase steadily to 0.90, 0.62, and 0.93, respectively. The curve of CDK for weighted *Chemprop* increases to 0.48 on the CHEMBL dataset. For PCAs, the curves for both *Chemprop* and weighted *Chemprop* show no clear correlations due to small data size. The jitter curves of *Chemprop* in most cases with lower *r*^2^ values reveal that low-quality data in the training datasets affect the model performance. Specifically, data curation poses a negative effect on *r*^2^ for some special datasets, for example, the CHEMBL dataset. This outcome may indicate that the actual data quality of this dataset should be higher than the value we set, and thus, the data may be polluted by other datasets, resulting in poor performance. When comparing *Chemprop* with original dataset and weighted *Chemprop* with curated dataset, the results in the left side of Fig. 4 shows no clear trends or gradation on both increasing training dataset size in x axis or increasing prediction dataset size on BPZ, BDZ, and BPZ&BDZ dataset in y axis. In the right side of Fig. 4 however we can confirm two trends from both x and y axis in our evaluation. Firstly on the x direction, the *r*^2^ value increases steadily when the data size of the training dataset increasing from AQUA with one thousand compounds to KINECT of hundred of thousands. Secondly on the y direction, the *r*^2^ value of BPZ&BDZ dataset with 31 compounds is larger than BPZ and BDZ in most cases on 7 datasets. What’s more, the *r*^2^ value of BPZ with 19 compounds is larger than that of BDZ with 12 compounds. Thus there is a clear gradation on increasing prediction dataset size on our curated dataset.

In Fig. 5, the *R*_*s*_ curves of BPU, BDZ, and BPU&BDZ converge to 0.59, 0.89, and 0.947, respectively, with increasing data size when using weighted *Chemprop*. The *R*_*s*_ value of PCAs and CDK increase to 0.58 and 0.63 on the CHEMBL dataset and decrease to 0.4 and −0.18 on the Kinect dataset, respectively. One can see that the Kinect dataset yields a negative performance on *R*_*s*_ when predicting the PCA and CDK values for both weighted *Chemprop* and *Chemprop*. The unstable *r*^2^ and *R*_*s*_ values around 0 for CDK confirm that the graph learning model of *Chemprop* fails to track the physicochemical features of PCAs in terms of solubility. From both Figs. 4, 5, weighted *Chemprop* demonstrates a clear prediction performance gradation on the BPU, BDZ, CDK and PCA molecules, whereas *Chemprop* with the original dataset does not.

The above comparison confirms that *r*^2^ and *R*_*s*_ values for CDK and PCA are noisy, these two datasets with 7 and 10 elements respectively, are too small to deliver a good comparison. However when given enough number of compounds, both the *r*^2^ and *R*_*s*_ value of BPU & BDZ datasets are high and above 0.9. As both *r*^2^ and *R*_*s*_ are used to evaluate the correlation coefficients of the predicted and observed values, it is not the absolute value of solubility value. We guess that intrinsic solubility and kinetic solubility can have different absolute solubility value but can still share the same tread in its correlation coefficients. Thus we didn’t distinguish between thermodynamic solubility and kinetic solubility in our training datasets (AQUA, PHYS, ESOL, OCHEM, AQSOL, CHEMBL, KINECT) and the test dataset (BPU, BDZ, CDK, and PCA). Note that, it is still recommended to avoid mixing kinetic and thermodynamic in one training dataset or test dataset. Larger dataset will be better for us to do this evaluation, but currently no other open data is available.

In terms of running time, predicting these 48 molecules, for example, with weighted *Chemprop* requires approximately 1.34 seconds in total or 0.028 seconds for each molecules on average with a single desktop computer as listed in Table 5. For QM-QSPR approaches such as the QM-based methods^2^, the calculation relies on a cloud infrastructure of millions of CPU cores; however, no running time can be recorded as their method is commercial and not publicly available. Thus, the availability of open-source methods and dramatically lower usage of computing resources are additional advantages of applying deep learning models.

**Table 5:** Statistical time-usage (averaged over 100 rounds) of predicting compounds in evaluation, ESOL, and AQSOL datasets with weighted *Chemprop* on three computers.

Desktop		Time Usage (in seconds)		
CPU	GPU	Evaluation (48)	ESOL (1128)	AQSOL (9982)
E3-1225 v6	—	1.28	8.11	86.56
E3-1225 v6	Quadro P400	1.34	8.49	86.07
Platinum 8180	—	0.70	9.98	107.93
Platinum 8180	GTX 1050Ti	0.61	8.27	86.28
Platinum 8180	Tesla T4	0.62	8.42	91.20

The number of molecules containing in these datasets are 48, 1311, 9982 respectively. The time-usage is measured in seconds. The efficiency of the prediction workload is about 4% on Tesla T4, 6% on GTX 1050Ti, and 9% on Quadro P400, thus the running time has limited relation with GPU cards for unsaturated workload.

To conclude, with seven collected large-scale aqueous solubility dataset and the proposed data curation methodology, seven high quality curated datasets with quality weights are generated. Deep learning methods including both *Chemprop* and *AttentiveFP* shares a dramatically increase on predictive accuracy measured in RMSE, which has been demonstrated in method section in details. More importantly, using these ensembled models with best RMSE, deep learning methods benefit from curated datasets, with a steady improvement in *r*^2^ and *R*_*s*_ when increasing training data volume. Deep learning methods also demonstrate a superior performance on *r*^2^ and comparable performance on *R*_*s*_ when predicting BPU and BDZ derivatives for leading compound optimization compared with the QM-QSPR approaches, such as^2^. A clear prediction performance rank demonstrating the capacity of deep learning methods on four series compounds is also illustrated by curated datasets. For example, deep learning methods do not function well on PCA and CDK derivatives, while the QM-QSPR approaches have not demonstrated their capacity. A clear advantage of deep learning approach is its running time, when predicting thousands of target compounds it takes only seconds on a common desktop computer whereas physics-based approach requires a large compute resources and takes a longrunning time.

## Discussion

Previously, both AI and drug design experts are focused on molecular property prediction. However they are interested in totally different issues, as we illustrated in Table 6. Enormous high quality data and high predictive accuracy on their own measurement standard are the main concern for the AI experts. Drug design experts are more interested in real world effects of the method itself. For example, how is the correlation coefficients in compound lead optimization, what’s the generalization ability on different series of in-house compounds, what’s the required computing resource and its running time on making prediction, and finally is it available or open-sourced for free application. This work is trying to bridge such gap with one of its sub-problem, aqueous solubility prediction.

**Table 6:** Difference of the issues concerned by AI experts and Drug design experts.

AI experts	Drug design experts
Data volume	Correlation coefficients in compound lead optimization
Data quality	Generalization ability on different series of compounds
Measurement standard	Computing resource and its running time
Predictive accuracy	Open source availability

Currently, the QM-QSPR approaches are the dominant techniques for aqueous solubility prediction in drug design. Several research works have demonstrated their improvement with AI techniques. However, with these continuous improvements in predictive accuracy achieved with AI, conservative drug design experts remain concerned about the real ability of deep learning in comparison with that of QM-QSPR approaches on their in-house datasets. This work contributes to resolve the concerned issues from both deep learning and drug design side. From the deep learning side, we increased the data volume of aqueous solubility datasets from thousand to hundreds of thousands of molecules, refined the data quality of the datasets with a data curation method, and finally improved the solubility predictive accuracy dramatically under the traditional measurement of RMSE. In terms of drug design side, this work is a milestone bridge that constructs a mechanism to compare QM-QSPR and deep learning approaches with state-of-the-art solubility evaluation datasets on correlation coefficients. Fortunately, the graph learning method of expanded *Chemprop* trained on a curated dataset has demonstrated a steady performance on correlation coefficients of *r*^2^ and *R*_*s*_ comparable to that of the QM-QSPR approaches, while using orders of magnitudes less compute resources and being available for public evaluation. The comparison also confirms that the generalization ability of deep learning approach is good on BPU andBDZ derivatives but still limited on PCA and CDK derivatives which demands further research effort on both sides.

This work also reveals a turning point in molecular property prediction where the deep learning and QM-QSPR approaches should be jointly co-developed. For example, topology-based graph learning and crystal-3D-structure-based deep learning may integrate both topology and crystal 3D features in solubility prediction with a promising accuracy improvement. One can also expand this work to other molecular properties to better understand natural phenomena with the help of both QM-QSPR and deep learning methods.

## Usage Notes

Reproducibility of the curation algorithm, training workflow and performance evaluation can be verifed by executing the scripts described in the README of our project SolCuration at https://github.com/Mengjintao/SolCuration. The code has been developed and tested using Python 3.7 on Linux operating system and is available under the BSD 3-Clause License. All the datasets are also provided in this repository for further research effort on this problem.

## Data Availability

The original, clean and curated dataset for the 7 selected data sources presented in this paper are publicly available on GitHub at https://github.com/Mengjintao/SolCuration and can be cited by^43^.

## Code availability

Python and C++ codes used to perform data curation, training workflow, and performance evaluation shown in this manuscript are publicly available on GitHub at https://github.com/Mengjintao/SolCuration or one can cite our code by^43^.
